# A Review on the Kenaf/Glass Hybrid Composites with Limitations on Mechanical and Low Velocity Impact Properties

**DOI:** 10.3390/polym12061285

**Published:** 2020-06-04

**Authors:** Seri Nur Zumaimi Ahmad Nadzri, Mohamed Thariq Hameed Sultan, Ain Umaira Md Shah, Syafiqah Nur Azrie Safri, Adi Azriff Basri

**Affiliations:** 1 Laboratory of Biocomposite Technology, Institute of Tropical Forestry and Forest Products (INTROP), UPM Serdang 43400, Selangor Darul Ehsan, Malaysia or seri_nurzumaimi@yahoo.com (S.N.Z.A.N.); ainumaira91@gmail.com (A.U.M.S.); snasafri@gmail.com (S.N.A.S.); 2 Department of Aerospace Engineering, Faculty of Engineering, Universiti Putra Malaysia, UPM Serdang 43400, Selangor Darul Ehsan, Malaysia; adiazriff@upm.edu.my; 3 Aerospace Malaysia Innovation Centre (944751-A), Prime Minister’s Department, MIGHT Partnership Hub, Jalan Impact, Cyberjaya 63000, Selangor Darul Ehsan, Malaysia

**Keywords:** kenaf fibre, glass fibre, hybrid composites, low velocity impact

## Abstract

Environmental awareness and trends to develop sustainable resources have directed much research attention towards kenaf fibre as an alternative reinforcement in composite manufacturing. Numerous studies have been conducted on kenaf and its hybrid composites. Most studies were conducted on kenaf/glass hybrid composites compared to other kenaf/synthetic hybrid composites. Similar with other materials, mechanical properties were the fundamental knowledge identified by the researcher. Limited studies conducted on other properties have restricted the use of kenaf composites to non-structural applications. To extend the potential of kenaf composites to automotive exterior or other critical applications, studies on impact properties can be a valuable contribution in the material field. This review discusses the mechanical and low velocity impact properties of kenaf/glass hybrid composites reported previously. Percentage loading of fibre, the angle of orientation in woven fibres and the chemical treatment applied to the fibre before compounding are the three major parameters that affect the mechanical and impact properties of the composites. This review provides insights into the mechanical and impact properties of kenaf/glass hybrid composites for future research.

## 1. Introduction

The usage of natural fibres such as kenaf in automotive applications has the potential advantage of reducing the weight of the vehicle which helps to minimise the fuel consumption, thus reducing the emission of harmful gases [1,2]. However, due to several drawbacks such as strength of natural fibres, the application of these materials was limited to interior parts of vehicles. In expanding the potential applications of natural fibre composites to the exterior parts, the use of synthetic fibre as a hybrid material had been the interest of researchers around the world [3,4]. Pertaining to the chances of sudden impact to the exterior parts of vehicles, low velocity impact analysis is another vital aspect that needs to be addressed besides the main mechanical properties [5,6]. Therefore, this review was conducted to give an overview of the two main properties of kenaf, glass and their hybrid composites, which were mechanical and low velocity impact properties. Kenaf and glass fibre composites were seen to have big potential in various applications based on numerous studies reported to date. 

The synthetic fibre such as glass fibre was found to enhance the mechanical properties of the composite [7]. Embedding woven E-glass as the outermost layer of three plied composites, also containing kenaf and jute, was reported to increase the tensile strength by more than 50% [8]. In the previous research, the tensile strength of bamboo/glass reinforcing polyester hybrid composite was 80.56 MPa compared to the tensile strength of bamboo reinforced polyester composite and bamboo/jute reinforced polyester hybrid composites, recorded as 46.67 and 60.32 MPa, respectively [9]. As reported in a previous study, the hybridization of hemp and glass composite presented the increasing trend of flexural strength changing from 97.5 to 101 MPa as the glass content in the composite increased from 0% to 15% [10].

Reducing the percentage of glass fibre in glass/polypropylene (PP) composites from 60% to 30% by replacing the reduced percentage with 30% twill woven bamboo fibres decreased the Charpy impact strength by less than 50%. However, in comparison with non-hybrid bamboo/PP composites, the glass/bamboo hybrid composites showed higher a Charpy impact strength, of 1129 J/m, compared to 530.9 J/m for the non-hybrid [11]. A composite with hybrid long fibres of both sugar palm and glass fibre, with a random orientation and 30:70 fibre ratio, respectively, in an epoxy matrix, showed its ability to withstand an impact energy up to 15 J in drop weight impact testing [12]. Various natural fibres have been used to be hybridised with glass fibres in an attempt to reduce the dependency on synthetic materials. On the other hand, the inclusion of glass fibre broadens the potential use of natural fibres in different applications.

This paper presents a short review on the mechanical and low velocity impact properties of kenaf/glass hybrid composites, to provide a reference for filling the gap in the respective research area. The aim is to enhance the potential of kenaf/glass hybrid composites to be used in automotive applications, based on their strength and impact properties.

## 2. Kenaf Plant

Kenaf plant, scientifically registered as *Hibiscus cannabinus L*., belongs to the family of Malvaceae. This herbaceous dicotyledonous plant grows during the warm season, similarly to okra and cotton plants, which belong to the same family of Malvaceae [13,14]. Table 1 shows the taxonomy of the kenaf plant. 

Kenaf plant was introduced in the 1970s in Malaysia as an industrial crop in Kelantan, Pahang and Terengganu [15]. Then, kenaf plantations have been identified as an alternative to tobacco plantations [16]. Kenaf plant became popular in the 1990s, when it turned out to be an alternative resource for composite fabrications, such as in particle board, medium-density fibre board (MDF), wood plastic composites (WPC) and many more applications [17]. Besides being one of the alternative raw materials for replacing wood fibre in the pulp and paper industries, kenaf plant is also traditionally used to make ropes, canvas, and sacks [18]. 

## 3. Extraction of Kenaf Fibres

**Figure 1:**
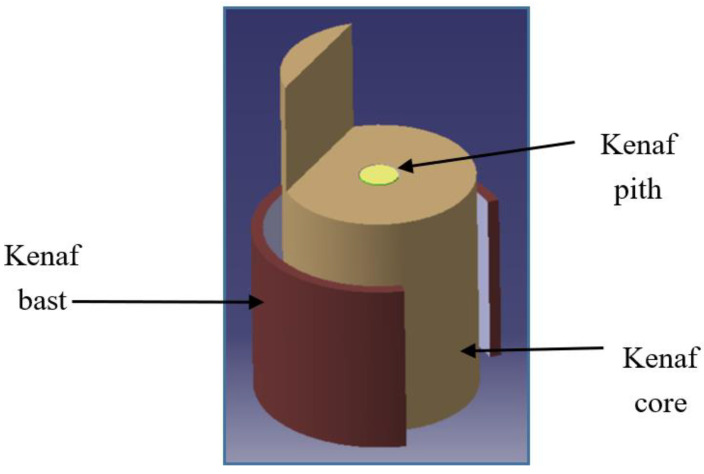
The illustration of kenaf stem.

In engineering applications, specifically in the composites field, kenaf fibres need to be extracted from the respective parts of the kenaf plant. It has been reported that approximately 40% of the kenaf plant stem can be transformed into fibres. This high percentage makes kenaf economically comparable to other plants, such as jute, hemp, and flax [13]. The short growing cycle of the kenaf plant, in the range of 150 to 180 days, and its low water requirement, make it more suitable to be used in composites and other applications [19,20]. Figure 1 shows the illustration of bast fibre, which is the outermost tissue layer of the kenaf stem, as well as the core and pith, which are located in the centre of the kenaf stem [21,22].

**Figure 2:**
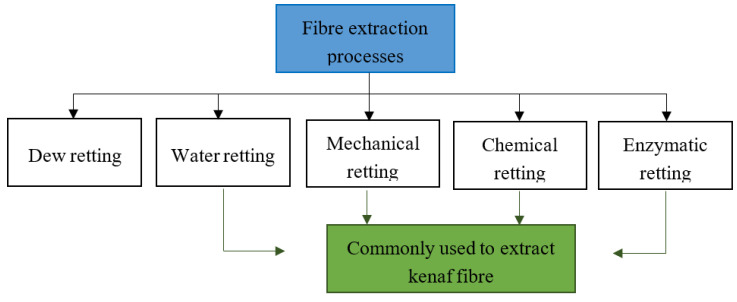
Different retting processes in extracting plant fibre.

The process of extracting plant fibres is also known as the retting or degumming process. There are a few retting methods applied to plant fibres reported to date, such as dew retting, water retting, enzymatic retting, chemical retting and mechanical retting [23]. Among them, the water, chemical, mechanical and enzymatic retting are the most commonly practiced processes in extracting kenaf fibres, as shown in Figure 2.

Compared to mechanical and chemical retting, water retting takes a longer time to complete the process, which is about 22 days, including washing, drying and combing the fibre. This method begins by submerging the kenaf plant into water, such as in a pond, river or tank. Microbial actions will separate the kenaf fibres within the pith (Figure 1). This process, however, will lead to minor water pollution and produce a strong odour because of anaerobic bacterial fermentation [24,25]. The water retting process will be continued by washing the fibre with clean water to remove unwanted substances. The cleaned fibres will be oven dried at 60 °C for 24 h until the fibre achieves constant weight [26]. A traditional method, such as air drying, can be used, depending on the surrounding humidity [27]. The combing process is carried out to disentangle the fibre, and at the same time to further extract single fibres from fibre bundles [28].

**Figure 3:**
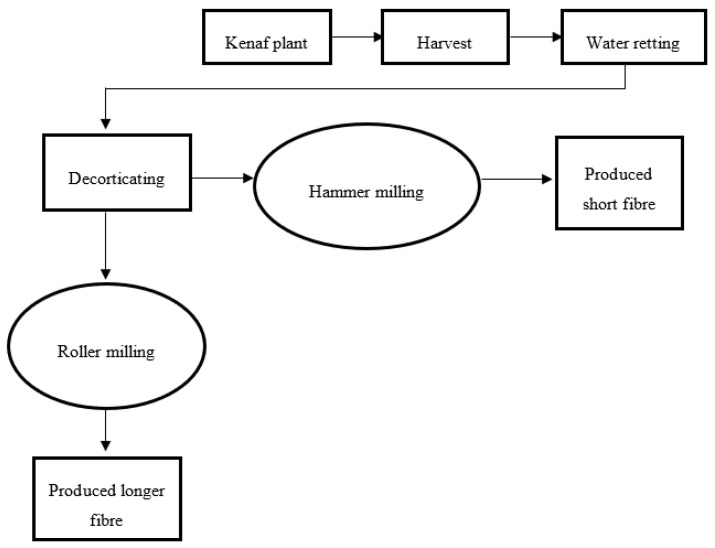
Fibre decortication process.

Mechanical retting, also called decortication, is the process of isolating the bast and the pith (Figure 1) using a decorticator. This method is easier, cheaper, and faster, compared to the other retting methods. Kenaf stalk will be immersed into water for at least 5 days before separating the fibre through the decorticating process [29]. Commonly used decorticating processes involve hammer milling and roller milling. Hammer milling is the process of beating the kenaf stem until the bast and core are separated. The small fibre obtained after the hammering process will be passed through the meshes inside the hammer milling machine. The roller milling machine will roll and crush the kenaf stalk, producing minimal damage to the fibre. Using this machine, longer fibre is achieved. The process of extracting fibre continues by washing, drying, and combing, similarly to the procedures in the water retting process, as explained earlier [25,29]. Figure 3 illustrates the fibre decortication process applied to extract kenaf fibre.

Physical retting, through steam explosion, is performed on the plant stalk before applying chemicals to soften the fibres. This process is eco-friendly and requires a shorter time to extract the fibres. Steam explosion is carried out at 0.5 MPa for 15 min, followed by alkali-oxygen treatment. These parameters have been suggested as the optimum pressure and time, respectively, to remove pectin, hemicellulose and lignin [24,30].

Chemical retting is the process where alkali hydrolysis takes place. The chemicals that are normally used are sodium hydroxide (NaOH), sodium benzoate (C_7_H_5_NaO_2_), and hydrogen peroxide (H_2_O_2_). Smooth and long consistent fibre can be produced through this process in a short period of time. It has been suggested that these chemicals should be used in concentrations below 1%, as a more concentrated alkaline solution could lead to the degradation of the tensile strength [24].

Enzymatic retting is based on a similar concept to that of water retting, which uses bacteria that produce pectic enzyme to extract the fibre by dissolving pectin. As biotechnology has advanced, this enzyme can be now produced in the laboratory and is commercially available. Enzymatic retting can be controlled, is efficient and most importantly environmentally friendly [31]. A suitable temperature range between 40 and 50 °C has been established based on the most favorable working conditions for the enzyme [26,32]. However, this process involves higher costs, compared to water retting. The later washing, drying and combing processes are all similar to the procedure described for other types of retting. 

## 4. Glass Fibre

Glass fibre is a commonly used synthetic fibre made from silicates, soda, clay, limestone, boric acid and various metallic oxides. All these constituents will undergo a heating process and will be refined in the narrow chamber [33]. Glass fibre is classified into several types, based on its main properties, and is denoted with letters C, D, E and S. C-glass fibre can be used in an acidic environment because it has the ability to resist corrosion. D-glass fibre has a low dielectric constant and it is commonly used in electrical applications. E-glass fibre is known as electrical glass, which is very good for electrical insulation. S-glass is a high strength glass fibre. It not only has high strength and stiffness, but also can be used in extreme conditions, such as in extreme temperatures or corrosive environments [34]. Table 2 shows the mechanical properties of different types of glass fibres. 

## 5. Mechanical Properties of Kenaf/Glass Hybrid Composites 

Kenaf fibre has low density and good mechanical properties. Replacing part of synthetic fibre such as glass fibre with kenaf fibre in a composite will reduce the structure’s weight and cost, and will be more environmentally friendly. The mechanical properties of kenaf and glass fibres are illustrated in Table 3.

Kenaf/glass hybrid composites with 90° orientations was reported to achieve higher tensile strength compared to 0° orientations with values of 69.86 and 49.27 MPa, respectively. The load applied in the direction parallel to the fibre contributed to the higher strength of 90° orientations kenaf/glass hybrid composites compared to the load applied perpendicular to the orientation of fibre [38]. The contribution of synthetic fibre, which is known for its strength and stiffness, was seen in the increment of tensile and flexural strength of kenaf/glass hybrid composites to 65.29 and 115.71 MPa compared to non-hybrid kenaf composites with 49.48 and 77.63 MPa, respectively [39].

Table 4 summarises the findings reported from studies on the mechanical properties of kenaf/glass fibre hybrid composites.

Chemical treatment using alkaline solution is the most commonly used due to its low cost and high effectiveness [41]. Kenaf fibre treated with 9% NaOH solution for 12 h presented the highest flexural strength of 93.3 MPa compared to 3% NaOH and untreated kenaf fibre which were 63.2 and 25.1 MPa, respectively. The adequate surface roughness of kenaf fibre treated with 9% NaOH improved the fibre matrix bonding, while a 3% NaOH concentration was not sufficient to remove the impurities on the surface of fibre [42]. In a different study, maintaining the percentage of kenaf fibre loading at 25% in two types of polymer matrix, epoxy and polyester, results in higher tensile strength for the epoxy-based composites with a value of 93.59 MPa compared to polyester-based composites at 86.54 MPa [43]. The fact that epoxy resin has better properties (such as high strength and low shrinkage) compared to polyester resin which is poor in adhesive and high cure shrinkage, indirectly contributes to the overall strength of composites [44].

In a research project that investigated the mechanical properties of different amounts of kenaf (K) layers in glass (G) fibre composites, one layer of kenaf as the core between three layers of glass fibre each at the top and bottom of the sandwich structure (3G/K/3G) presented the highest tensile strength compared to 3G/2K/3G and 3G/3K/3G, which had two and three layers of kenaf as the core in the same sequences. The study suggested that the increment in the number of kenaf layers will reduce the tensile strength due to the low strength of kenaf fibre [45]. For different types of kenaf fibres, increasing the fibre loading from 10% to 40% increased the flexural strength of composites accordingly, whereas further increasing the fibre loading to 50% decreased the flexural strength due to the saturated mixture of fibre in the polymer matrix. The saturated mixture had decreased the ability of polymer to hold the fibre tightly, thus could not sustain the load applied to the whole structure [46]. The fibre size is also one of the important factors in determining the mechanical properties of composite. It was reported that 30% kenaf fibre in 20 mesh size had a higher tensile strength of 16 MPa compared to 40 mesh kenaf fibre at 13.6 MPa. A larger surface area of 20 mesh filler was suggested to enhance the surface contact and bonding between fibre and matrix [47].

## 6. Low Velocity Impact Properties of Kenaf Composites

Reaction such as rebounding, penetration and perforation will happen during a low velocity impact event [48]. The damage of the specimen can be analysed through the force against displacement graph. Closed loop graph curve indicated that the impactor rebounded when it hit the surface of the specimen [49]. The damages that happened during the low velocity impact event included the matrix cracking, delamination, fibre failure and penetration [50]. Matrix cracking is the initial stage of damage due to low velocity impact. The number of cracks will increase when a larger external load is applied leading to another failure which is delamination [51]. Delamination was found to be induced by matrix cracking when the high transverse shear stresses at the neighboring impacted matrix surface and later develops into a weak interfacial bond which develops into fibre fracture and fibre pullout [52,53]. The phenomena of shear cracking and bending cracking are often characterizations of pine tree damage. Pine tree damage usually exhibits on the stiff-thick composite and reserved pine tree damage exhibits on the flexible-thin composite [54,55]. Perforation is the damage when the impactor completely penetrated the specimen. The opened loop curve in force against displacement graph determined that the specimen experienced penetration by the impactor [49].

Researchers have reported that the highest energy absorbed by kenaf/epoxy composites was of 2.83 J at 15% fibre content. Meanwhile, a kenaf polyester composite absorbed the maximum energy of 4.53 J at 25% fibre content [56]. Kenaf fibre reinforced polyvinyl butyral (PVB) showed an increasing trend of impact strength when the fibre content was increased from 10% to 20% and 30%, while the highest impact strength result was achieved at 40% fibre content. However, the impact energy strength of the composite started decreasing at 50% and 60% fibre content [57]. The decreasing trend of the impact strength graph when the fibre content was at 50% and 60% also presented in the research on the hybridization of kenaf fibre reinforced recycled polypropylene/polyethelene (r-PP/PE) [58]. Fibre content is an important influencing factor applied not only to kenaf reinforced polymer composites, but also to kenaf hybrid composites. A study showed that the 50:50 fibre ratio in bamboo/kenaf hybrid composites led to the highest impact strength result, which was 45 J/m, compared to the only kenaf reinforced epoxy composite and to the 30:70 fibre ratio in bamboo-kenaf hybrid composites, which recorded 40.6 and 37.8 J/m, respectively, for impact strength [59].

In a study, three different fibre arrangements were used to fabricate kenaf/polyester composites, namely, perpendicular, anisotropic and isotropic. Pure polyester was used as a control. The impact strength of the obtained materials was tested on an Izod impact tester. Pure polyester showed the lowest result of impact strength, which is 1.42 kJ/m^2^. Meanwhile, the highest impact strength result was recorded for the kenaf/polyester composite with the anisotropic fibre arrangement of 6.68 kJ/m^2^ [60]. Fibre size also affects the impact energy absorbed by the composites [60]. A study focusing on kenaf fibre reinforced thermoplastic polyurethane (TPU) composites showed that the impact strength values of the materials prepared with the smallest size of kenaf fibre (<125 µm), medium size of kenaf fibre (125–300 µm) and the largest size of kenaf fibre (300–425 µm) were 2.1, 2.76 and 2.97 kJ/m^2^, respectively [61].

Different types of matrix gave different impact strength results for woven kenaf fibre composites. In a previous study, the same type of fibre, namely woven kenaf fibre, and the same impact energy, but different fibre orientations, of 0°/90° and 45°/−45°, have been used to fabricate composites using three different types of matrix, specifically, epoxy, unsaturated polyester, and vinyl ester. Woven kenaf fibre reinforced unsaturated polyester showed the highest energy absorbed for both types of fibre orientation [57].

Kenaf fibre tends to absorb moisture because of its hydrophilic nature. Therefore, chemical treatment is applied to modify the fibre surface by removing the hydroxyl groups and impurities from the fibre [62]. A few chemical treatments can be performed to modify the kenaf fibre surface, such as alkaline treatment, silane treatment and a combination of alkaline and silane treatment [63]. The treatment using NaOH can be summarised in the chemical equation below [63]:

Fibre-OH + NaOH = Fibre + Na^+^O^−^ + H_2_O + impurities (1)

The untreated fibre presented low impact strength due to the weak interfacial bond between fibre and matrix. The elimination of the hemicellulose, wax and –OH bonding during the fibre treatment enhanced the bonding between fibre and matrix. Treated kenaf fibre gave a higher impact strength to the composites, compared to the untreated one. This is because the NaOH solution that had been used in treating kenaf fibre improved the properties of kenaf-reinforced thermoplastic polyurethane composites [64]. Izod impact testing revealed that an untreated kenaf reinforced polyester composite showed the lowest value of impact strength, which was 2.61 kJ/m^2^. Two different concentrations of NaOH solution, of 6% and 9%, had been used to treat kenaf fibre for 12 h. The impact strength of the composite comprising fibre treated with 6% NaOH concentration was 15.77 kJ/m^2^, while that of the composite with fibre treated with 9% NaOH concentration was 6.92 kJ/m^2^ [49]. Kenaf fibre treated with propionic anhydride gave better impact strength results, of 7.7 kJ/m^2^, compared to kenaf fibre treated with succinic anhydride and untreated fibre, which recorded 7.3 and 5.4 kJ/m^2^, respectively [62]. A previous study compared the impact strength of untreated and treated woven kenaf-banana hybrid composites. The authors concluded that the impact strength of the untreated woven kenaf-banana hybrid composite was 23 kJ/m^2^, that of the alkali treated woven kenaf-banana material was 26 kJ/m^2^ and that of the material treated with sodium lauryl sulfate (SLS) was 28 kJ/m^2^ [65]. The findings of reported research studies about the effects of kenaf fibre treatment on the impact strength of the prepared composites are summarised in Table 5. Enzymatic treatment has been also used to modify the surface of fibre. In previous research, kenaf fibre treated with laccase enzyme showed a 26.31 J/m^2^ impact strength, while untreated fibre showed a 22.51 J/m^2^ impact strength [66].

Hybrid banana/kenaf/banana reinforced epoxy composites were evaluated by using the Charpy test and the maximum impact energy was found to be 12 J. This is the highest impact energy achieved in this study, among other hybrids, such as kenaf/kenaf/kenaf and neem/kenaf/neem composites, which reached values of 4 and 10 J, respectively [65]. The hybridization of kenaf fibre with synthetic fibre such as Kevlar enhanced the impact strength performance of the hybrid composite [67]. In a previous study, the highest impact strength of kenaf/Kevlar hybrid composite that been recorded was 50.1 kJ/m^2^ which belonged to the Kevlar/kenaf/Kevlar/kenaf hybrid composite. This study also concluded that the number of fibre layers and fibre sequences became factors that affected the impact properties of the kenaf/Kevlar hybrid [68].

## 7. Low Velocity Impact of Glass Composites

Previous research reported that E-glass composites are more resistant to impact compared to C-glass, specifically, that smaller damage at a higher peak force was found on E-glass composites at the same impact energy level [69]. In terms of weaving pattern, woven E-glass fibre gives better mechanical properties, compared to chopped strand fibre mat. It was reported that woven E-glass fibre has an impact strength of 415 MPa, while chopped strand E-glass fibre presented 189 MPa [70].

Fibre content and fibre arrangement affect the impact properties of not only natural fibre composites, but also of glass fibre composites. A study showed that the maximum impact energy absorbed by composites made from one layer of plain-weave glass fibre and two layers of chopped strand glass mats, with 30% fibre content, was 112.105 kJ/m^2^, while that of composites fabricated from two layers of plain-weave glass fibre and one layer of chopped strand glass mat, with 22% fibre content, was 77.141 kJ/m^2^ [70]. Another study reported that a glass wool fibre reinforced epoxy material with a 40% fibre weight gave the highest impact strength value, which was 0.0862 J/m^2^, compared to 30% and 50% glass wool fibre weight composites, which reached 0.0652 and 0.0421 J/m^2^, respectively [71].

## 8. Low Velocity Impact of Kenaf/Glass Hybrid Composites

The impact strength properties of hybrid composites have been found to be affected by several factors, including volume fraction, fibre matrix adhesion, fibre orientation, fibre length, stress transfer and the thickness of the composites [72]. The reported research findings on kenaf fibre and glass fibre hybrid composites are tabulated in Table 6.

The composite fabricated with a 90° kenaf/glass fibre orientation had a higher impact strength compared to the one with a 0° fibre orientation, namely, of 6.66 and 6 J, respectively. Due to its better impact strength, it was suggested that the hybrid composite can be used in structural applications [38]. Previous work reported that twisted neem/kenaf/neem composites, with a fibre orientation of 90°/45°/90°, embedded with glass fibre at the outermost top and bottom layers, recorded the maximum impact strength of 12.2 J, which was higher than the values achieved by neem/kenaf/neem composites with fibre orientations of 0°/0°/0° and 0°/90°/0° (11.23 and 11.64 J, respectively) [65].

At 9 J of impact energy, kenaf/glass hybrid composites with 3 mm thickness showed a major crack length of 52.92 mm, while kenaf/epoxy composites reached 100.61 mm for major crack length. The smaller damage on the kenaf/glass hybrid composites indicated that embedded woven glass hybridised with kenaf led to stronger impact resistance. Meanwhile, the glass/epoxy composite showed 16.02 mm radius of damage [79]. In another study, 75% glass fibre and 25% kenaf fibre composites showed almost similar results to those achieved for 100% glass fibre. The hybrid composites were selected to undergo low velocity testing. The hybrid composite with 10 layers of glass and kenaf fibre could absorb an impact energy of up to 40 J [49].

## 9. Conclusions

Kenaf fibre has great potential in automotive application as a reinforcement fibre, since it is light in weight, eco-friendly, low-cost and has good mechanical properties. Reinforcing kenaf fibre with glass fibre is one of the methods to enhance kenaf fibre composites because glass fibre has better mechanical and impact properties than kenaf fibre. There are several factors that affect the mechanical and impact properties of the kenaf/glass hybrid composites. Previous studies show that the optimum fibre content and fibre orientation were between 30% and 40% and 90°, where it can resist a higher impact strength and have better mechanical properties. Fibre surface modification also helps to improve the properties of kenaf/glass hybrid composites. The number of layers and the thickness of the composite also influences its mechanical and impact strength properties. From the review conducted, it can be suggested that kenaf/glass hybrid composites have higher tensile strength up to 85 MPa compared to kenaf composites. Other than that, kenaf/glass hybrid composite can also withstand low velocity impact energy up to 12 J. The mechanical and impact properties of kenaf/glass hybrid composites discussed in this manuscript show its potential for use in automotive applications. It was also found that limited studies were done on the mechanical and impact properties of the kenaf/glass hybrid composites, and further investigations were needed to maximise the potential of kenaf/glass hybrid composites. 

## Figures and Tables

**Table 1:** Kenaf plant′s taxonomy.

Kingdom	Plantae
Class	Magnoliopsida
Order	Malvales
Family	Malvaceae
Genus	Hibiscus
Species	*Hibiscus cannabinus L.*

**Table 2:** Mechanical properties of glass fibre types C, D, E and S.

	C-Glass	D-Glass	E-Glass	S-Glass	Ref.
Density (g/cm^3^)	2.56	2.11	2.54	2.53	[34]
Tensile strength (MPa)	3300	2500	3400	4600	
Young’s Modulus (MPa)	69	55	72	89	
Elongation (%)	4.8	4.5	4.7	5.2	

**Table 3:** General mechanical properties of kenaf fibre and glass fibre.

	Kenaf	Glass
Density (g/m^3^)	1.45	2.55
Tensile strength (MPa)	930	3400
Elastic modulus (GPa)	53	71
Elongation at break (%)	1.6	3.4
References	[18,35]	[36,37]

**Table 4:** Reported research on mechanical properties of kenaf/glass fibre hybrid composites.

Materials	Mechanical Properties		Ref.
	Tensile	Flexural	
90° fibre orientation of kenaf + chopped strand glass fibre reinforced epoxy	69.86 MPa	162.566 MPa	[38]
kenaf fibre yarn + woven glass fibre reinforced epoxy	65.29 MPa	115.71 MPa	[39]
Woven kenaf fibre + glass fibre mat reinforced unsaturated polyester	85.49 MPa	124.07 MPa	[40]

**Table 5:** Previous studies on different kenaf fibre treatment.

Composites		Parameters	Impact Strength	Ref.
Kenaf + polyester	Chemical treatment	Untreated	2.61 kJ/m^2^	[42]
		Treated with 6% NaOH	15.77 kJ/m^2^	
		Treated with 9% NaOH	6.92 kJ/m^2^	
Kenaf + polyester		Untreated	5.4 kJ/m^2^	[62]
		Propionic anhydride	7.7 kJ/m^2^	
		Succinic anhydride	7.3 kJ/m^2^	
Kenaf/banana + unsaturated polyester		Untreated	23 kJ/m^2^	[65]
		Treated with alkali	26 kJ/m^2^	
		Treated with sodium lauryl sulfate	28 kJ/m^2^	
Kenaf + recycled polypropylene	Enzymatic treatment	Untreated	22.51 J/m^2^	[66]
		Treated with laccase enzyme	26.13 J/m^2^	

**Table 6:** Previous studies on kenaf/glass fibre hybrid composites.

Kenaf Fibre	Glass Fibre	Matrix	Fabrication Method	Ref.
Woven kenaf fiber	Woven E-glass fiber	Epoxy	Hand lay-up	[73]
Twisted long kenaf fiber	Glass fiber	Epoxy	Hand lay-up	[74]
Kenaf fiber (direction 0°, 90°)	Glass fiber direction (0°, 90°)	Epoxy	Hand lay-up	[38]
Twisted kenaf fiber	Woven glass fiber	Epoxy	Vacuum pump Compression moulding	[75]
Kenaf fibre	Woven fibre glass	Polyester	Hand lay-up Cold press	[76]
Kenaf mat (20 cm × 20 cm)	Glass fiber (20 cm × 20 cm)	Unsaturated polyester	Hand lay-up Compression	[77]
Kenaf fibre	Glass fibre	Unsaturated polyester	Cold pressure hand lay-up	[78]
Non- woven kenaf mat	E-glass fibre	Polypropylene	Compression moulding	[79]
